# Phenotype Prediction of Pathogenic Nonsynonymous Single Nucleotide Polymorphisms in *WFS1*

**DOI:** 10.1038/srep14731

**Published:** 2015-10-05

**Authors:** Xuli Qian, Luyang Qin, Guangqian Xing, Xin Cao

**Affiliations:** 1 Department of Biotechnology, School of Basic Medical Science, Nanjing Medical University, Nanjing, P.R. China; 2 Department of Otolaryngology, the First Affiliated Hospital of Nanjing Medical University, Nanjing, P.R. China

## Abstract

Wolfram syndrome (WS) is a rare, progressive, neurodegenerative disorder that has an autosomal recessive pattern of inheritance. The gene for WS, wolfram syndrome 1 gene (*WFS1*), is located on human chromosome 4p16.1 and encodes a transmembrane protein. To date, approximately 230 mutations in *WFS1* have been confirmed, in which nonsynonymous single nucleotide polymorphisms (nsSNPs) are the most common forms of genetic variation. Nonetheless, there is poor knowledge on the relationship between SNP genotype and phenotype in other nsSNPs of the *WFS1* gene. Here, we analysed 395 nsSNPs associated with the *WFS1* gene using different computational methods and identified 20 nsSNPs to be potentially pathogenic. Furthermore, to identify the amino acid distributions and significances of pathogenic nsSNPs in the protein of *WFS1*, its transmembrane domain was constructed by the TMHMM server, which suggested that mutations outside of the TMhelix could have more effects on protein function. The predicted pathogenic mutations for the nsSNPs of the *WFS1* gene provide an excellent guide for screening pathogenic mutations.

Wolfram syndrome (WS) (MIM 222300), also known as DIDMOAD (diabetes insipidus, insulin-deficient diabetes mellitus, optic atrophy and deafness), is a rare neurodegenerative disorder of autosomal recessive inheritance, characterised by diabetes insipidus, insulin-deficient diabetes mellitus, optic atrophy and deafness. Of these symptoms, diabetes mellitus is the most common manifestation of WS with a median onset age of 6 years^1^ and always presents before the age of 16^2^. The prevalence of WS is approximately 1/700,000 individuals in the UK, and 1/100,000 individuals in North America^3^. Since the first report for WS by Wolfram and Wagener in 1938^4^, progressively more cases have been observed. Many studies have been performed to investigate the genetic basis of this hereditary disease and have identified that loss-of-function mutations in the *WFS1* gene are the main cause of the syndrome^5^.

*WFS1*, located on human chromosome 4p16.1, is composed of eight exons, of which only the first exon is a noncoding exon, and most mutations in *WFS1* have been identified in exon 8 but also in exons 3, 4, 5 and 6^6^,^7^,^8^. *WFS1* encodes the protein wolframin, which is abundantly expressed in pancreas, brain, heart, and muscle and is thought to be a novel endoplasmic reticulum (ER) calcium channel or a regulator of channel activity^9^,^10^. Additionally, wolframin appears to be involved in membrane trafficking, protein processing^11^, regulation of intracellular Ca^2+^ homeostasis^12^ and β-cell dysfunction^13^,^14^. Mutations in the *WFS1* gene may result in instability and a significantly reduced half-life of wolframin in the endoplasmic reticulum and then may cause disease^15^.

To date, approximately 230 mutations in *WFS1* have been reported (https://lovd.euro-wabb.org/home.php?select_db=WFS1). Although nsSNPs are the most common form of genetic variation in these mutations, the relationship between the genotype and phenotype of other nsSNPs in the *WFS1* gene is unclear. Given the large number of nsSNPs in the *WFS1* gene, it is expensive and time-consuming to experimentally explore the functional effects of these SNPs. The prediction of the phenotypic effects of nsSNPs based on different computational methods has become a well-known methodology^16^,^17^, and several research articles have cited its effectiveness in identifying deleterious, disease-related mutations^18^,^19^. In those methods, predicting pathogenic nsSNPs is based on identifying structural and functional damaging properties. This study will facilitate the investigation of the role of nsSNPs in *WFS1* and identify pathogenic nsSNPs associated with the *WFS1* gene based on different computational methods. Among these methods, the prediction of deleterious and damaging nsSNPs was performed by SIFT and PolyPhen-2. A support vector machine (SVM) along with the SIFT algorithm, PhD-SNP and MutPred were used to detect disease-associated nsSNPs. In addition, to identify the amino acid distributions and significances of pathogenic nsSNPs in the protein of *WFS1*, we constructed the transmembrane domain by the TMHMM server v2.0.

## Results

### SNP dataset from databases

The nsSNPs were collected from the NCBI dbSNP, HGMD, Deafness Variation Databases and the Locus Specific Database, in which the NCBI dbSNP database was the primary source, containing approximately 1,500 SNPs, and the other three were as supplemental. After filtering, a total of 395 nsSNPs were identified.

### NsSNP prediction results of *WFS1*

To identify deleterious mutations from the nsSNPs in the *WFS1* gene, the SIFT and PolyPhen-2 server were used to predict whether the mutations were deleterious/damaging. The SIFT server was used to calculate the tolerance index of all 395 collected nsSNPs with evolutionary conservation analysis, and a SIFT score value of <0.05 was considered to be deleterious. Meanwhile, we subjected all 395 nsSNPs to the PolyPhen-2 structure-based analysis server to further analyze the effects of amino acid substitutions (AAS) on the structures and functions. Of the 395 nsSNPs in the *WFS1* gene, 174 nsSNPs were predicted to be deleterious by SIFT and the remaining nsSNPs were tolerated except for nonsense mutations for which SIFT provided no score. Among these deleterious nsSNPs, 32 mutations (P7L, G154A, W314R, P346L, Y351C, S353C, R375C, E394V, E394K, S430L, S430W, Y528D, P533S, A684V, A684T, A684G, C690R, C690G, G695V, Y699H, Y699C, Y699S, G702S, G702D, R708C, N714T, G736R, G736D, G736S, G834S, L842F and P885L) were reported to be highly deleterious with SIFT scores of 0.000. Obviously, in these highly deleterious nsSNPs, the mutation frequencies in the amino acid loci 394, 430, 684, 690, 699, 702 and 736 were higher than other loci. In PolyPhen-2, 235 nsSNPs were predicted to be damaging to protein structure and function, of which 89 mutations were predicted to be highly deleterious with PolyPhen-2 scores of 1.000. A total of 156 nsSNPs were predicted to be deleterious and damaging by both SIFT and PolyPhen-2 (Table 1) after excluding all nonsense mutations. Additionally, of these 156 nsSNPs, 28 nsSNPs (P346L, Y351C, S353C, R375C, E394V, E394K, S430L, S430W, Y528D, P533S, Y669H, Y669C, Y669S, A684T, A684G , A684V, C690R, C690G, G695V, G702D, G702S, R708C, G736D, G736R, G736S, G834S, L842F and P885L) were predicted to be highly deleterious and damaging by both algorithms with SIFT scores of 0.000 and PolyPhen-2 scores of 1 (Table 1).

For further study, we used PhD-SNP and MutPred to investigate whether these 156 filtered deleterious and damaging nsSNPs were associated with disease. PhD-SNP is optimised to classify disease-causing point mutations from the given datasets, and MutPred is also a web application tool developed to classify an AAS as either disease-associated or neutral in humans but also predicts the molecular cause of disease/deleterious AASs. Of the 156 nsSNPs, 97 diseased-associated nsSNPs were predicted by PhD-SNP and 91 nsSNPs were predicted to be disease-associated by MutPred tools. But it is worth noting that some of the 28 mutations with scores of 0.000 for SIFT and 1.000 for Polyphen-2 in Table 1 like P346L, Y351C, G834S or L842F were not predicted as diseased-associated by both PhD-SNP and MutPred, this might be because the loci of these amino acid were conserved, but the mutants on these loci could not cause the molecular changes or affect the whole protein structure. Finally, 70 nsSNPs were predicted to be diseased-associated using both PhD-SNP and MutPred, in which the numbers of mutations predicted as very confident hypotheses, confident hypotheses and actionable hypotheses were 16, 33 and 21, respectively. The most common changes in the molecular mechanisms in the mutants predicted by MutPred were gains or losses of helixes and sheets. Representative diseased-associated nsSNPs and the corresponding AAS of nsSNPs in the *WFS1* gene are provided in Table 2. After inspecting these mutations in their reference sources, most of the nsSNPs predicted have also been reported, demonstrating that the nsSNPs predicted were credible from multiple computational methods. Finally, we predicted 20 mutations (F329I, S353C, R375H, R375C, E394K, F439C, R517P, L594R, P607L, S662P, T665I, R732C, R732H, G736D, Y739D, C742R, R832C, R859W, R868C and A874T) to be potentially pathogenic mutations, and 50 other mutations had been previously published or cited (Table 2).

**Figure 1:**
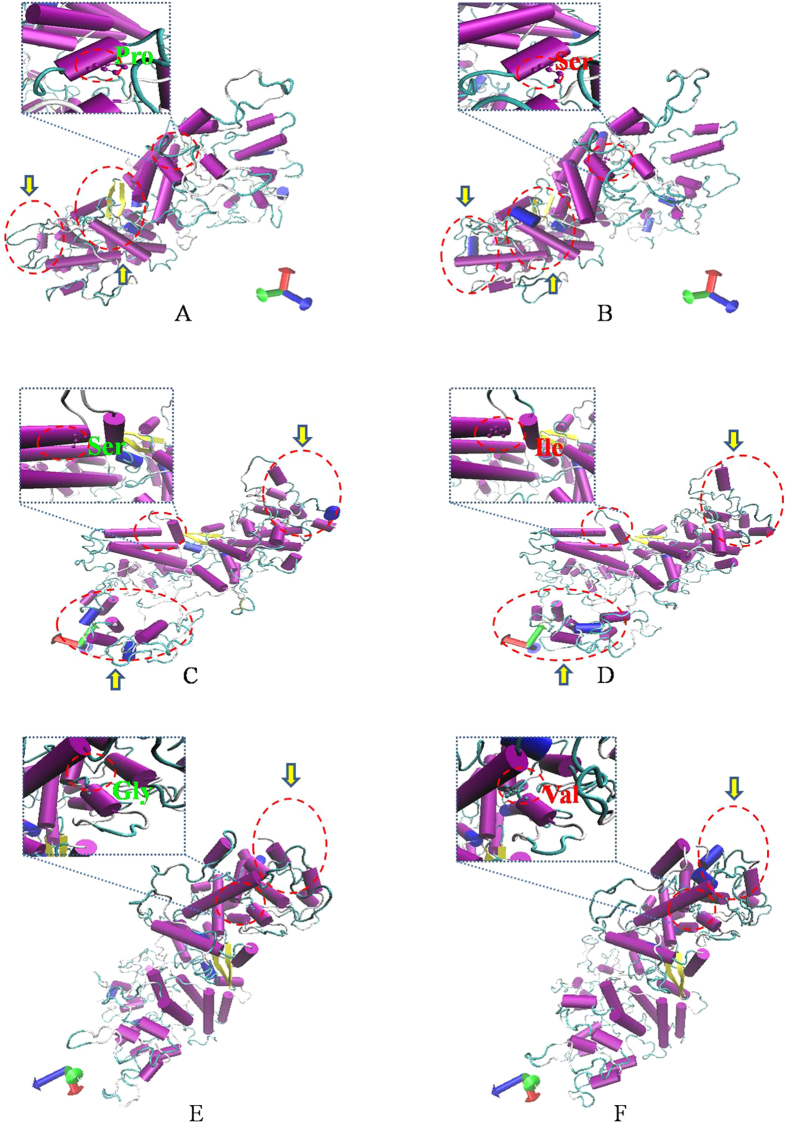
Protein structure predicted by the SWISS-MODEL server. (**A,B**) indicate the changes between wild type and mutant wolframin with the amino acid change P292S, (**C,D**) depict the structural changes between wild type and mutant S443I, and E and F illustrate the effects of G695V. (**A,C,E**) are protein structures of the wild type wolframin, and (**B,D,F**) are structures of the mutant proteins (created by SWISS-MODEL and illustrated with VMD). The arrows in yellow and the circles in red indicate the differences between the wild type and the mutant.

**Figure 2:**
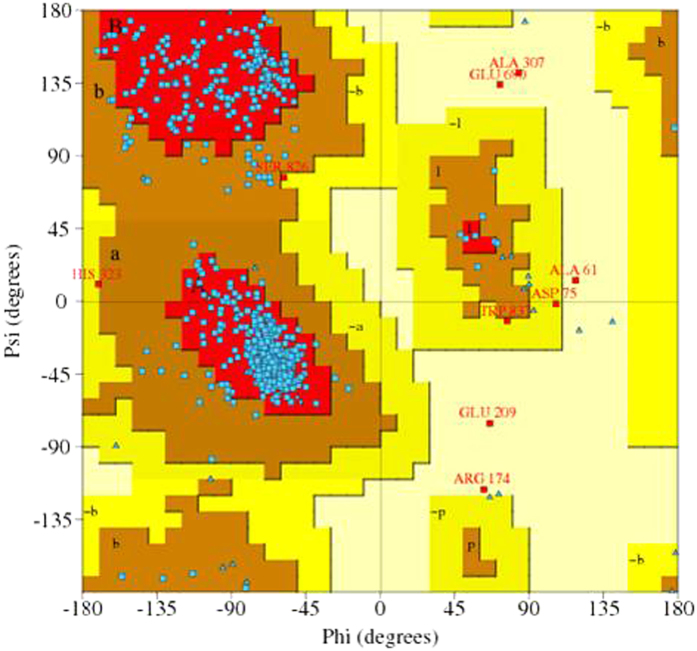
Ramachandran Plot of the wild type wolframin protein structure evaluated by PROCHECK.

Additionally, to better understand how the pathogenic nsSNPs affect protein conformation and result in disease states, we constructed wild type and mutant proteins via the Robetta and SWISS-MODEL tools (Fig. 1, Supplementary file 1–4). And the geometric evaluations of the modeled 3D structure were performed using PROCHECK by calculating the Ramachandran plot (Fig. 2). The wild type protein showed 99.4% of residues in most favoured and allowed region and the overall average of G factors was 0.27 which showed the structure was usual. In this step, we randomly selected three predicted nsSNPs (P292S, S443I and G695V) that have been reported to be pathogenic^6^,^20^,^21^ and compared the structures between the wild type and mutant proteins. We observed that after mutation, not only did the amino acid change, but it also affected the entire protein structure. All of the three protein structures (P292S, S443I and G695V) representing different mutations gained or lost some α-helixes, suggesting a potential molecular mechanism resulting in WS.

### Amino acid distribution in the transmembrane domain

**Figure 3:**
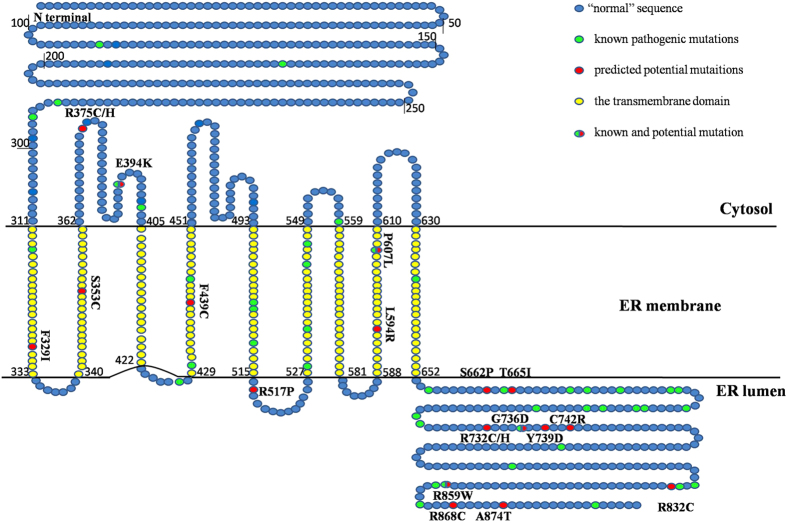
Transmembrane domain structure of wolframin and its distribution of mutations^22^. The 70 predicted pathogenic mutations are highlighted with green/red coloured circles compared to “normal” sequence with blue circles 
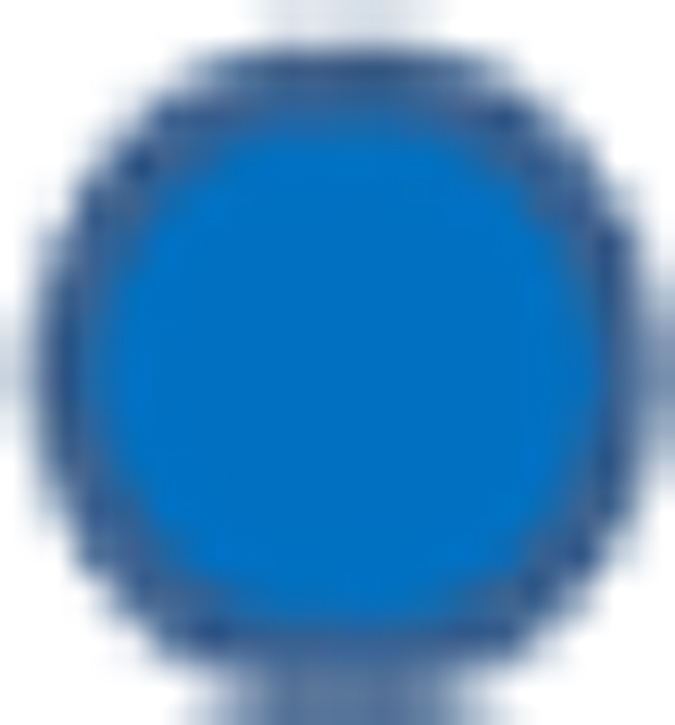
. The 50 known pathogenic mutations are depicted in green 
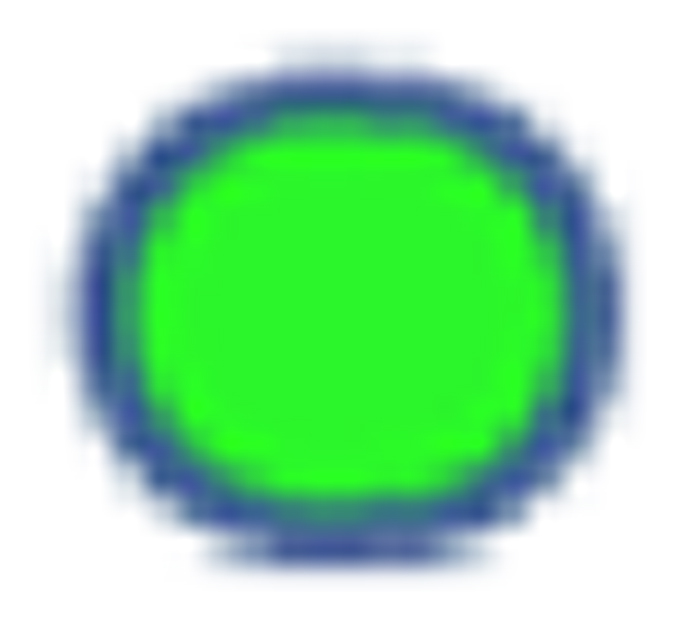
 and the 20 predicted potentially pathogenic mutations are in red 
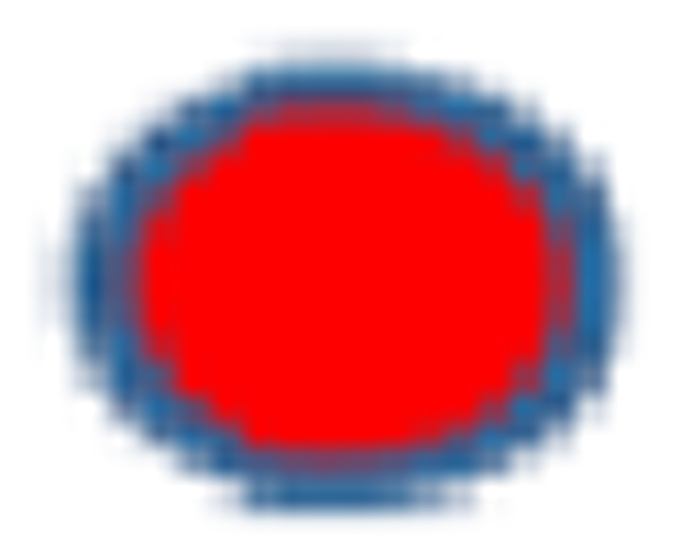
. The transmembrane domain is depicted in yellow 
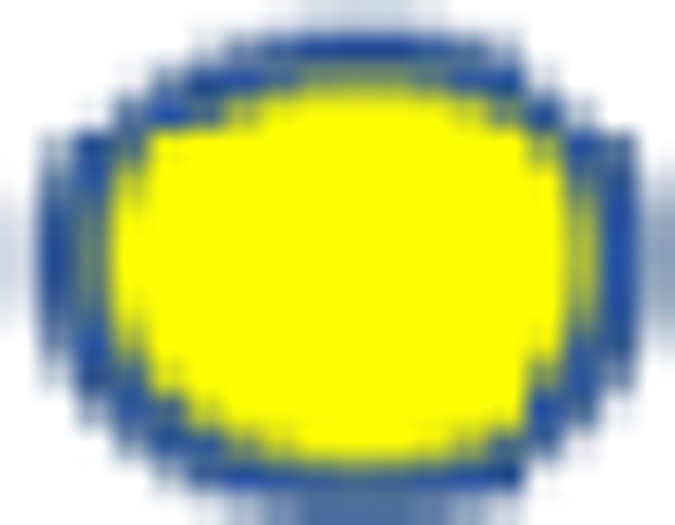
. The circle with green and red 
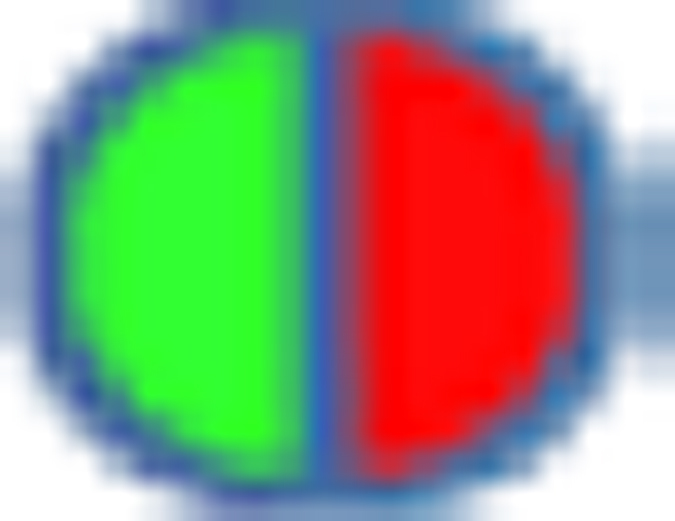
 denotes that the locus has a known and predicted mutation.

To elucidate the amino acid distributions and significances of predicted pathogenic nsSNPs in wolframin, we constructed its transmembrane domain using the TMHMM server v2.0 (Fig. 3). In this analysis, the transmembrane domain of wolframin was divided into 9 TMhelixes, with each TMhelix being approximately 23-amino acids long. Except for the third and seventh TMhelix, 18 pathogenic mutations were distributed across the other seven TMhelixes, accounting for 25.71% of all 70 pathogenic mutations, of which 13 were previously known. Notably, most pathogenic mutations in our study were not located in the transmembrane domain but in the C-terminal domain of wolframin (Table 3). In all 70 pathogenic mutations, approximately 52 were not located in the TMhelix (74.29%), 39 of which were located in the C-terminal domain. Thirty-seven pathogenic mutations have been previously reported in the 52 mutations not located in the TMhelix, and only 15 mutations were predicted to be potentially pathogenic.

## Discussion

WS is a rare autosomal recessive disorder with a number of loss-of-function mutations of the *WFS1*, both within and between most affected patients/families. Wide tissue distribution of wolframin and many mutations in *WFS1* resulting in WS may contribute to different phenotypes. Growing evidences have presented many clinical signs and possible correlations between the genotype and the development of the neurologic manifestations, the age at onset of diabetes mellitus, hearing defects, and diabetes insipidus in WS on the cohort of WS patients^22^,^23^. So far, although a large number of variants of the *WFS1* gene have been identified, novel mutations are continuously found in this gene. Furthermore, the pathogenic role of different mutations, polymorphisms and sequencing variants of the gene remains largely unknown. Phenotypic prediction of the effects of nsSNPs might identify meaningful changes in genes that alter protein function to induce phenotypic consequences. The sheer number of SNPs in online databases provides an abundant resource to predict the phenotypic effects of nsSNPs, and known pathogenic mutations from the literature provide us an opportunity to inspect prediction accuracy, which indicates whether the relationships between nsSNP prediction results and known pathogenic mutations are confirmed by *in vivo and in vitro* experiments.

In the present study, we predicted 20 potentially pathogenic mutations and 50 known pathogenic mutations using *in silico* methods, and combined the results of the most common changes by MutPred and the predictions of the three protein structures by the SWISS-MODEL to determine that the most probable mutational effects causing WS might be the gains or losses of α-helixes. It is worth to consider that some predicted pathogenic nsSNPs have been confirmed by *in vitro* functional studies and genetic analysis for WS families, which could indirectly verify the accuracy of our methods. For example, p.P724L(c.2171C>T) and p.G695V(c.2084G>T) of *WFS1* have been reported to lead to WS and which cause the formation of detergent-insoluble aggregates of wolframin when was expressed in COS-7 cells^24^; the p.A684V(c.2051C>T) and p.L511P (c.1532T>C) were ectopically expressed in HEK293 cells which showed reduced protein levels compared to wild type wolframin, strongly indicating that the mutation is disease-causing^21^,^25^. Meanwhile, by direct DNA sequencing and linkage analysis, p.L804P (c.2411T>C) and p.R859P (c.2576G>C) were identified after screening the entire coding region of the *WFS1* gene in a Chinese WS family and in a US family with the nonsyndromic hearing loss, respectively^26^,^27^.

*WFS1* spanning approximately 33.4 kb of genomic DNA, consists of eight exons and produces a peptide product which is 890-amino acid long (wolframin). The amino acid distribution results of wolframin suggest that wolframin contains 9 transmembrane domains. These results are consistent with the previous research which provides experimental evidence that wolframin contains 9 transmembrane segments and is embedded in the membrane in an Ncyt/Clum topology^15^. However, the prediction for wolframin available at UniProt database gives 11 transmembrane domains (http://www.uniprot.org/uniprot/O76024) (Table 4), and the difference between the two predicted results was mainly in the TMhelix 5, TMhelix 6 and TMhelix 11. In our result, the 493–515 amino acids are located in TMhelix5; while in UniProt, this region has been divided into TMhelix 5 and TMhelix 6 domains, respectively; the 653–890 amino acids have also been predicted as two TMhelixes in the same way in the UniProt. With reference to most researches, the wolframin were considered as 9 transmembrane domains with some evidences, and this is due to the differences in the execution of algorithm. Additionally, our results also indicate that the mutations outside of the TMhelix could have more pronounced functional effects, especially in the C-terminal with 39 predicted mutations. Many of the reported missense mutations are located in the C-terminal hydrophilic part of the protein^15^, and the experiments also support these predictions. Just as de Heredia *et al*. found that besides the transmembrane domains, the mutations identified in WS patients also concentrate in the last 100 amino acids in the C-terminal^1^. Using yeast two-hybrid analysis, Zatyka *et al*. identified that the C-terminal domain of wolframin, which is positioned in the ER lumen, bound the C-terminal domain (amino acids 652–890) of the ER-localized Na^+^/K^+^ ATPase beta-1 subunit (ATP1B1)^28^. And the Na^+^/K^+^ ATPase deficiency has a crucial role in apoptosis and in neural degenerative disease which can be induced by mutations in *WFS1*, leading to the development of WS^29^.

In summary, we used extensive functional and structural level analyses to predict potentially pathogenic mutations for nsSNPs in the *WFS1* gene and analysed the amino acid distributions of wolframin to provide a guide for screening pathogenic mutations and investigating the function of wolframin. Furthermore, we provide information for predicting the effects of nsSNPs in genes encoding transmembrane proteins and for further research in variant effect prediction.

## Materials and Methods

### Dataset collection

NsSNP datasets of the *WFS1* gene were obtained from the NCBI dbSNP database (http://www.ncbi.nlm.nih.gov/projects/SNP/)^30^, HGMD (http://www.hgmd.cf.ac.uk/ac)^31^, Deafness Variation Database (http://deafnessvariationdatabase.org) and the Locus Specific Database (https://lovd.euro-wabb.org/home.php?select_db=WFS1). The amino acid sequence of wolframin was retrieved from the UniProt database (http://www.uniprot.org/). Data for the *WFS1* gene were collected from Entrez Gene on the NCBI web site (http://www.ncbi.nlm.nih.gov/genbank/), and the literature search was performed using PubMed, Science Direct, and Web of Science.

### Filtering and mining of nsSNPs

Because SNPs from the databases were not initially nsSNPs, we needed to perform some manual filtering. In this process, we eliminated SNPs in 3′ or 5′UTRs and synonymous SNPs. For prediction and analysis, SNP ID, gene name, protein accession, amino acid residue 1 (wild type), amino acid position, and amino acid residue 2 (missense) for all nsSNPs were collected from the NCBI dbSNP database, HGMD, and Deafness Variation Databases.

### Predicting the phenotype of nsSNPs with the SIFT and PolyPhen-2 tools

After filtering the nsSNPs, we predicted their functional effects with the SIFT (http://sift-dna.org) and PolyPhen-2 (http://genetics.bwh.harvard.edu/pph2/) tools. In SIFT server, a highly conserved position is more likely to be deleterious with a SIFT score <0.05, whereas a tolerant mutation will have a SIFT score >0.05^32^,^33^. PolyPhen-2 extracts various sequence- and structure-based features of the substitution site and inputs them into a probabilistic classifier based on a given AAS and protein accession. The mutation is appraised qualitatively, as benign, possibly damaging, or most likely damaging^34^.

### Identifying disease-associated nsSNPs using the PhD-SNP and MutPred tools

PhD-SNP (http://snps.biofold.org/phd-snp) and MutPred (http://mutpred.mutdb.org/) were based on a support vector machine (SVM) and the SIFT algorithm. To PhD-SNP, in briefly, after inputting the protein sequence, position and new residue, the substitution from the wild type residue to the mutant is encoded in a 20-element vector that is −1 in position relative to the wild type residue, 1 in the position relative to the mutant residues and 0 in the remaining 18 positions. Next, a second 20-element vector encoding the sequence environment is constructed to report the occurrence of residues in a window of 19 residues around the mutated residue. With this supervised learning approach, a given mutation is classified as disease or neutral^35^,^36^.

MutPred is based on SIFT scores, the gain or loss of 14 different structural and functional properties. Two important scores are contained in the output of MutPred: a general score (g), and top 5 property score (p). The general score (g) indicates the probability that the AAS is deleterious/disease-associated, whereas the top 5 property score (p) is the *P*-value that indicates whether certain structural and functional properties are affected. The combinations of high general scores and low property scores are referred to as actionable hypotheses, confident hypotheses, and very confident hypotheses^37^.

### Protein structure prediction of pathogenic nsSNPs via Robetta and SWISS-MODEL tools

As the structure of wolframin is not available and there is not suitable template for modelling, so we choose the Robetta server (http://robetta.bakerlab.org/) to construct the protein structure. The Robetta server is a full chain protein structure prediction server for *ab initio* and comparative modeling, and the SWISS-MODEL (http://swissmodel.expasy.org/) is a fully automated, dedicated protein structure homology-modelling server^38^,^39^. The amino acid sequence of wolframin was retrieved from NCBI (accession number: NP_005996.2). 3D-structure of wolframin was performed using Robetta server. And the mutant proteins were constructed by SWISS-MODEL with the template performed using Robetta server (Sup.file S). The quality of the modelled structure of native and mutant protein was evaluated by the PROCHECK (http://services.mbi.ucla.edu/SAVES/).

### Analysis of the transmembrane domain by the TMHMM server v2.0

TMHMM server v2.0 (http://www.cbs.dtu.dk/services/TMHMM/), based on a hidden Markov model (HMM) with an architecture that corresponds closely to the biological system, is a membrane protein topology prediction method. Compared with other servers, TMHMM server v2.0, which is thought to be currently the best performing transmembrane prediction program, can model and predict the location and orientation of alpha helices in membrane-spanning proteins with high accuracy^40^.

## Additional Information

**How to cite this article**: Qian, X. *et al*. Phenotype Prediction of Pathogenic Nonsynonymous Single Nucleotide Polymorphisms in *WFS1*. *Sci. Rep*. **5**, 14731; doi: 10.1038/srep14731 (2015).

## Supplementary Material

Supplementary Information

Supplementary PDB files

## Figures and Tables

**Table 1:** Deleterious and damaging nsSNPs of *WFS1* prioritised using SIFT and PolyPhen-2 scores.

Amino Acid Change	Nucleotide Variation	SIFT Score	PolyPhen-2 Score	SNP ID*
R24H	G/A	0.011	0.999	rs71524364
T104I	C/T	0.021	0.992	
G107E	G/A	0.004	1	rs71530914
G107R	G/A	0.003	1	*WFS1*_00227
Y110N	T/A	0.023	0.999	CM050353
D118A	A/C	0.004	0.999	rs71524349
A126T	G/A	0.007	1	rs145639028
G154A	G/C	0	0.996	rs71530927
T156M	C/T	0.002	1	
D171N	G/A	0.049	0.953	
R177P	G/C	0.010	1	CM083208
A198V	C/T	0.047	0.875	rs142687752
E202G	A/G	0.043	0.998	*WFS1*_00230
D211N	G/A	0.017	0.813	rs138682654
R228H	G/A	0.037	1	rs150771247
E273K	G/A	0.018	0.904	rs142428158
P292S	C/T	0.008	1	CM992981
I296S	T/G	0.003	0.688	CM992982
W314R	T/A	0	0.999	*WFS1*_00229
L327I	C/A	0.013	1	rs71537678
F329I	T/A	0.031	0.99	rs188848517
**P346L**	**C/T**	**0**	**1**	**CM073420**
F350V	T/G	0.045	0.999	
**Y351C**	**A/G**	**0**	**1**	**rs181988441**
**S353C**	**C/G**	**0**	**1**	**rs143547567**
C360Y	G/A	0.001	0.999	rs147157374
T361I	C/T	0.002	1	*WFS1*_00075
**R375C**	**C/T**	**0**	**1**	**rs200095753**
R375H	G/A	0.003	1	rs142671083
T378N	C/A	0.007	0.999	*WFS1*_00097
D389E	T/G	0.007	0.978	rs201282601
**E394K**	**G/A**	**0**	**1**	**rs373146435**
**E394V**	**A/T**	**0**	**1**	**rs146563951**
L402P	T/C	0.001	1	CM112216
H407R	A/G	0.010	0.684	rs140407862
V412A	T/C	0.021	0.981	rs144951440
F417S	T/C	0.002	0.95	rs111570388
I427S	T/G	0.005	0.903	CM073419
**S430L**	**C/T**	**0**	**1**	***WFS1*****_00218**
**S430W**	**C/G**	**0**	**1**	***WFS1*****_00194**
L432V	C/G	0.027	1	rs35031397
F439C	T/G	0.002	0.913	rs141585847
S443I	G/T	0.002	0.997	CM015195
T455M	C/T	0.027	1	rs139361521
R456C	C/T	0.010	0.689	rs144452795
E462G	A/G	0.016	0.99	rs398123066
E462G	A/G	0.016	0.99	
C505Y	G/A	0.001	0.998	CM031397
L506R	T/G	0.003	0.95	CM043878
L511P	T/C	0.001	0.949	
Y513S	A/C	0.036	0.98	
R517H	G/A	0.024	0.986	rs150394063
R517P	G/C	0.022	0.904	
M518I	G/A	0.013	0.978	rs138232538
A519V	C/T	0.047	1	rs201557396
**Y528D**	**T/G**	**0**	**1**	**CM087003**
**P533S**	**C/T**	**0**	**1**	**rs146132083**
C537Y	G/A	0.003	0.999	rs199910987
L543R	T/G	0.003	1	CM031400
V545M	G/A	0.038	0.992	rs201993978
V546D	T/A	0.004	0.999	CM031401
R558C	C/T	0.001	1	rs199946797
R558H	G/A	0.002	1	CM031402
A575G	C/G	0.018	0.528	rs71524360
G576S	G/A	0.031	0.882	rs1805069
V582M	G/A	0.009	0.916	rs377677092
R587W	C/T	0.005	0.999	rs138968466
L594R	T/G	0.001	0.999	rs200288171
A602E	C/A	0.011	0.74	rs2230720
A602G	C/G	0.001	0.74	
P607L	C/T	0.040	0.999	rs373862003
P607R	C/G	0.010	1	CM033825
R611C	C/T	0.008	0.999	rs144993516
L637P	T/C	0.002	1	*WFS1*_00215
T641M	C/T	0.018	0.985	rs376626985
R653C	C/T	0.007	1	rs201064551
E655G	A/G	0.006	0.999	CM024439
E655K	G/A	0.015	0.995	CM108408
S662P	T/C	0.004	1	rs376341411
L664R	T/G	0.001	1	CM090453
T665I	C/T	0.002	0.976	
T665N	C/A	0.005	0.544	rs138258392
T665P	A/C	0.004	0.544	rs369656458
**Y669C**	**A/G**	**0**	**1**	**CM983479**
**Y669H**	**T/C**	**0**	**1**	**CM072120**
**Y669S**	**A/C**	**0**	**1**	**CM090454**
L672P	T/C	0.026	0.998	CM056420
G674E	G/A	0.029	1	CM020990
G674R	G/A	0.024	1	rs200672755
G674V	G/T	0.013	1	CM020991
R676C	C/T	0.030	1	rs201623184
W678L	G/T	0.008	0.999	CM073425
**A684G**	**C/G**	**0**	**1**	
**A684T**	**G/A**	**0**	**1**	
**A684V**	**C/T**	**0**	**1**	**rs387906930**
R685C	C/T	0.003	1	rs112967046
R685P	G/C	0.023	0.999	CM081852
R685P	G/C	0.023	0.999	
I688T	T/C	0.002	0.999	
**C690G**	**T/G**	**0**	**1**	**CM087004**
**C690R**	**T/C**	**0**	**1**	**CM992988**
**G695V**	**G/T**	**0**	**1**	**rs28937891**
T699M	C/T	0.001	1	rs28937894
W700C	G/T	0.001	1	CM992989
**G702D**	**G/A**	**0**	**1**	**CM090455**
**G702S**	**G/A**	**0**	**1**	**rs71532862**
R703C	C/T	0.024	1	rs201888856
K705N	G/C	0.032	0.997	CM032680
**R708C**	**C/T**	**0**	**1**	**rs200099217**
R708H	G/A	0.003	1	rs369062548
D713G	A/G	0.012	0.999	rs143280847
N714T	A/C	0	0.998	rs397517196
L723P	T/C	0.001	1	
P724L	C/T	0.002	1	rs28937890
P724S	C/T	0.043	1	
R732C	C/T	0.007	1	rs71526458
R732H	G/A	0.018	1	rs149013740
**G736D**	**G/A**	**0**	**1**	**rs71530912**
**G736R**	**G/C**	**0**	**1**	
**G736S**	**G/A**	**0**	**1**	**rs71532864**
Y739D	T/G	0.006	1	rs367737581
C742R	T/C	0.010	1	rs71532865
C742W	C/G	0.002	1	rs71532866
R756C	C/T	0.002	1	rs138127684
A761V	C/T	0.031	0.818	rs71526459
H763P	A/C	0.014	0.995	
D771G	A/G	0.011	1	CM015267
D771H	G/C	0.003	1	CM052942
R772C	C/T	0.005	1	rs149540655
E776V	A/T	0.001	1	rs56002719
G780R	G/C	0.046	0.989	CM012813
G780S	G/A	0.049	0.896	rs387906931
R791C	C/T	0.019	0.982	rs200528166
K800E	A/G	0.038	0.958	rs55674815
L804P	T/C	0.001	1	*WFS1*_00226
S807R	A/C	0.012	0.973	CM020992
E809K	G/A	0.042	0.999	rs71539673
R818C	C/T	0.014	1	rs35932623
L829P	T/C	0.001	1	rs104893883
G831D	G/A	0.012	1	rs28937895
R832C	C/T	0.010	1	rs148089728
**G834S**	**G/A**	**0**	**1**	**rs398124214**
**L842F**	**C/T**	**0**	**1**	**rs71530915**
A844T	G/A	0.047	0.973	CM053436
A844V	C/T	0.036	0.999	rs200192011
R859P	G/C	0.004	1	CM052943
R859W	C/T	0.001	1	rs372298367
H860D	C/G	0.007	0.96	CM043881
I863M	C/G	0.003	0.977	rs71524393
E864K	G/A	0.045	1	rs74315205
R868C	C/T	0.008	1	rs148611943
R868H	G/A	0.031	1	rs56393026
A874T	G/A	0.006	1	rs200775335
K876T	A/C	0.006	0.98	rs144900514
**P885L**	**C/T**	**0**	**1**	**rs372855769**
A889V	C/T	0.024	0.855	rs147934586

^*^In the SNP ID column, the nsSNPs with the prefix “rs” are from dbSNP, and those with the prefix “CM” and “*WFS1*_” are from HGMD and Locus Specific Database, respectively, and the remaining with no SNP ID are in the Deafness Variation Database. The nsSNPs highlighted in bold are predicted to be highly deleterious and damaging, with a SIFT score of 0, and PolyPhen-2 score of 1.

**Table 2:** Diseased-associated nsSNPs of *WFS1* predicted using the PhD-SNP and MutPred servers.

Amino Acid Change	*g* Value	*p* Value	Molecular Change	Prediction Reliability	SNP ID*	Reported or not
Y110N	0.849	0.0133	Gain of disorder	Confident Hypotheses	CM050353	Y^41^
R177P	0.817	0.0021	Loss of MoRF binding	Very Confident Hypotheses	CM083208	Y^42^
P292S	0.942	0.0093	Gain of helix	Very Confident Hypotheses	CM992981	Y^20^
I296S	0.867	0.0051	Gain of loop	Very Confident Hypotheses	CM992982	Y^20^
W314R	0.884	0.0162	Gain of methylation at W314	Confident Hypotheses	*WFS1*_00229	Y^43^
**F329I**	**0.774**	**0.0344**	**Gain of sheet**	**Actionable Hypotheses**	**rs188848517**	**N**
**S353C**	**0.502**	**0.0266**	**Gain of sheet**	**Actionable Hypotheses**	**rs143547567**	**N**
**R375H**	**0.670**	**0.0444**	**Loss of helix**	**Actionable Hypotheses**	**rs142671083**	**N**
**R375C**	**0.669**	**0.0444**	**Loss of helix**	**Actionable Hypotheses**	**rs200095753**	**N**
E394V	0.811	0.0425	Gain of helix	Confident Hypotheses	rs146563951	Y^44^
**E394K**	**0.826**	**0.0176**	**Gain of methylation at E394**	**Confident Hypotheses**	**rs373146435**	**N**
L402P	0.679	0.0215	Gain of relative solvent accessibility	Actionable Hypotheses	CM112216	Y^23^
I427S	0.828	0.0082	Gain of disorder	Very Confident Hypotheses	CM073419	Y^45^
S430L	0.793	0.0203	Loss of loop	Confident Hypotheses	*WFS1*_00218	Y^22^
S430W	0.790	0.0266	Gain of sheet	Confident Hypotheses	*WFS1*_00194	Y^23^
**F439C**	**0.835**	**0.0357**	**Loss of sheet**	**Confident Hypotheses**	**rs141585847**	**N**
S443I	0.836	0.0221	Gain of sheet	Confident Hypotheses	CM015195	Y^21^
C505Y	0.975	0.0062	Loss of catalytic residue at P504	Very Confident Hypotheses	CM031397	Y^46^
L506R	0.858	0.0196	Loss of helix	Confident Hypotheses	CM043878	Y^47^
L511P	0.748	0.0016	Gain of sheet	Actionable Hypotheses		Y^25^
**R517P**	**0.534**	**0.0072**	**Loss of helix**	**Actionable Hypotheses**		**N**
Y528D	0.939	0.0037	Loss of sheet	Very Confident Hypotheses	CM087003	Y^48^
P533S	0.886	0.0228	Loss of sheet	Confident Hypotheses	rs146132083	Y^44^
L543R	0.768	0.0228	Loss of sheet	Actionable Hypotheses	CM031400	Y^46^
V546D	0.828	0.0037	Loss of sheet	Very Confident Hypotheses	CM031401	Y^46^
R558C	0.890	0.0296	Loss of methylation at R558	Confident Hypotheses	rs199946797	Y^49^
R558H	0.950	0.0296	Loss of methylation at R558	Confident Hypotheses	CM031402	Y^46^
**L594R**	**0.688**	**0.0344**	**Gain of sheet**	**Actionable Hypotheses**	**rs200288171**	**N**
**P607L**	**0.748**	**0.0022**	**Gain of helix**	**Actionable Hypotheses**	**rs373862003**	**N**
P607R	0.954	0.0005	Gain of MoRF binding	Very Confident Hypotheses	CM033825	Y^50^
L637P	0.683	0.0072	Loss of helix	Actionable Hypotheses	*WFS1*_00215	Y^51^
E655G	0.756	0.0187	Loss of solvent accessibility	Actionable Hypotheses	CM024439	Y^44^
E655K	0.811	0.0049	Gain of MoRF binding	Very Confident Hypotheses	CM108408	Y^52^
**S662P**	**0.816**	**0.0312**	**Gain of loop**	**Confident Hypotheses**	**rs376341411**	**N**
L664R	0.926	0.0090	Gain of MoRF binding	Very Confident Hypotheses	CM090453	Y^53^
**T665I**	**0.821**	**0.0117**	**Gain of helix**	**Confident Hypotheses**		**N**
L672P	0.874	0.0076	Loss of helix	Very Confident Hypotheses	CM056420	Y^54^
G674R	0.964	0.0328	Gain of MoRF binding	Confident Hypotheses	rs200672755	Y^55^
G674V	0.958	0.0325	Gain of helix	Confident Hypotheses	CM020991	Y^56^
W678L	0.933	0.0132	Loss of catalytic residue at A677	Confident Hypotheses	CM073425	Y^57^
A684V	0.755	0.0104	Loss of helix	Actionable Hypotheses	rs387906930	Y^21^
R685P	0.859	0.0033	Loss of helix	Very Confident Hypotheses		Y^58^
C690R	0.945	0.0008	Gain of MoRF binding	Very Confident Hypotheses	CM992988	Y^20^
C690G	0.955	0.0115	Gain of disorder	Confident Hypotheses	CM087004	Y^48^
G695V	0.911	0.0036	Gain of sheet	Very Confident Hypotheses	rs28937891	Y^6^
H696Y	0.764	0.0390	Gain of sheet	Actionable Hypotheses	*WFS1*_00098	Y^59^
W700C	0.942	0.0157	Loss of MoRF binding	Confident Hypotheses	CM992989	Y^20^
G702S	0.887	0.0315	Loss of sheet	Confident Hypotheses	rs71532862	Y^23^
G702D	0.96	0.0315	Loss of sheet	Confident Hypotheses	CM090455	Y^53^
R708C	0.921	0.0182	Loss of MoRF binding	Confident Hypotheses	rs200099217	Y^21^
L723P	0.731	0.0045	Gain of loop	Actionable Hypotheses		Y^23^
P724L	0.926	0.0336	Loss of catalyticresi due at P724	Confident Hypotheses	rs28937890	Y^6^
**R732H**	**0.855**	**0.0444**	**Loss of helix**	**Confident Hypotheses**	**rs149013740**	**N**
**R732C**	**0.848**	**0.0376**	**Loss of helix**	**Confident Hypotheses**	**rs71526458**	**N**
**G736D**	**0.934**	**0.0425**	**Gain of helix**	**Confident Hypotheses**	**rs71530912**	**N**
G736R	0.965	0.0117	Gain of helix	Confident Hypotheses		Y^60^
**Y739D**	**0.736**	**0.0332**	**Gain of disorder**	**Actionable Hypotheses**	**rs367737581**	**N**
**C742R**	**0.814**	**0.013**	**Gain of disorder**	**Confident Hypotheses**	**rs71532865**	**N**
E776V	0.939	0.050	Gain of MoRF binding	Confident Hypotheses	rs56002719	Y^47^
L804P	0.768	0.0063	Loss of sheet	Actionable Hypotheses	*WFS1*_00226	Y^26^
L829P	0.928	0.0079	Gain of loop	Very Confident Hypotheses	rs104893883	Y^61^
G831D	0.923	0.0143	Gain of helix	Confident Hypotheses	rs28937895	Y^61^
**R832C**	**0.505**	**0.0228**	**Loss of sheet**	**Actionable Hypotheses**	**rs148089728**	**N**
**R859W**	**0.596**	**0.0152**	**Loss of disorder**	**Actionable Hypotheses**	**rs372298367**	**N**
R859P	0.853	0.0315	Loss of sheet	Confident Hypotheses	CM052943	Y^27^
H860D	0.769	0.0104	Loss of sheet	Actionable Hypotheses	CM043881	Y^47^
E864K	0.901	0.0016	Gain of MoRF binding	Very Confident Hypotheses	rs74315205	Y^62^
**R868C**	**0.843**	**0.0179**	**Loss of disorder**	**Confident Hypotheses**	**rs148611943**	**N**
**A874T**	**0.769**	**0.0061**	**Gain of sheet**	**Actionable Hypotheses**	**rs200775335**	**N**
P885L	0.953	0.0117	Gain of helix	Confident Hypotheses	rs372855769	Y^20^

^*^In the SNP ID column, the nsSNPs with the prefix “rs” are from dbSNP, and those with the prefix “CM” and “*WFS1*_” are from HGMD and Locus Specific Database, respectively, and the remaining with no SNP ID are in the Deafness Variation Database.The nsSNPs highlighted in bold are potential pathogenic nsSNPs which have not been reported.

**Table 3:** NsSNP distributions of the transmembrane domain of wolframin from the TMHMM server.

Distribution of Transmembrane Domain	Range of Amino Acid	Number of Reported Pathogenic nsSNPs	Number of Predicted Pathogenic nsSNPs	Total Number of nsSNPs in Each Domain	Ratio of Each Domain (%)
Outside	1–310	4	0	4	5.714
TMhelix 1	311–333	1	1	2	2.857
Inside	334–339	0	0	0	0
TMhelix 2	340–362	0	1	1	1.429
Outside	363–404	2	3	5	7.142
TMhelix 3	405–422	0	0	0	0
Inside	423–428	1	0	1	1.429
TMhelix 4	429–451	3	1	4	5.714
Outside	452–492	0	0	0	0
TMhelix 5	493–515	3	0	3	4.286
Inside	516–526	0	1	1	1.429
TMhelix 6	527–549	4	0	4	5.714
Outside	550–558	2	0	2	2.857
TMhelix 7	559–581	0	0	0	0
Inside	582–587	0	0	0	0
TMhelix 8	588–610	1	2	3	4.286
Outside	611–629	0	0	0	0
TMhelix 9	630–652	1	0	1	1.429
Inside*	**653–890**	28	11	39	**55.714**
Total	890-amino acids	50	20	70	100

^*^The domain highlighted in bold is the distribution of the C terminal domain.

**Table 4:** The prediction results to the transmembrane domain of wolframin from the TMHMM server and UniProt database.

TMHMM server		UniProt database	
Distribution of Transmembrane Domain	Range of Amino Acid	Distribution of Transmembrane Domain	Range of Amino Acid
Outside	1–310	Outside	1–313
TMhelix-1	311–333	TMhelix-1	314–334
Inside	334–339	Inside	335–339
TMhelix-2	340–362	TMhelix-2	340–360
Outside	363–404	Outside	361–401
TMhelix-3	405–422	TMhelix-3	402–422
Inside	423–428	Inside	423–426
TMhelix-4	429–451	TMhelix-4	427–447
Outside	452–492	Outside	448–464
**TMhelix-5**	493–515	**TMhelix-5**	465–485
		Inside	486–495
		**TMhelix-6**	496–516
Inside	516–526	Outside	517–528
TMhelix-6	527–549	TMhelix-7	529–549
Outside	550–558	Inside	550–562
TMhelix-7	559–581	TMhelix-8	563–583
Inside	582–587	Outside	584–588
TMhelix-8	588–610	TMhelix-9	589–609
Outside	611–629	Inside	610–631
TMhelix-9	630–652	TMhelix-10	632–652
Inside*	**653–890**	Topological domain	**653–869**
		**TMhelix-11**	**870–890**
Total	890-amino acids	Total	890-amino acids

^*^The domains highlighted in bold are the distributions of the C terminal domain.
