# Supplementary material for: Phenotype Prediction of Pathogenic Nonsynonymous Single Nucleotide Polymorphisms in WFS1
Source: Sci Rep. 2015 Oct 5;5:14731. doi: 10.1038/srep14731 (PMC4592972; doi:10.1038/srep14731)
Supplement: Supplementary Information [file srep14731-s1.doc]

***Supplementary information***

**Phenotype Prediction of Pathogenic Nonsynonymous Single Nucleotide Polymorphisms in *WFS1***

**Xuli Qian^1^,** **Luyang Qin^1^, Guangqian Xing^2^, Xin Cao^1^**

**Affiliations:**

^1^Department of Biotechnology, School of Basic Medical Science, Nanjing Medical University, Nanjing, P.R. China

^2^Department of Otolaryngology, the First Affiliated Hospital of Nanjing Medical University,

Nanjing, P.R. China

**Correspondence to:** Xin Cao **(**email: [caoxin@njmu.edu.cn](mailto:caoxin@njmu.edu.cn)**)**.

**This supplementary information includes:**

**Supplementary file 1**∣ The protein structure of wild type wolframin predicted by the SWISS-MODEL server (wild type-wolframin.pdb).

**Supplementary file 2**∣ The protein structure of mutant wolframin predicted by the SWISS-MODEL server (model P292S.pdb).

**Supplementary file 3**∣ The protein structure of mutant wolframin predicted by the SWISS-MODEL server (model S443I.pdb).

**Supplementary file 4**∣ The protein structure of mutant wolframin predicted by the SWISS-MODEL server (model G695V.pdb).

These supplementary files could be opened by Swiss-Pdb viewer (<http://spdbv.vital-it.ch/>), RasMol (<http://www.openrasmol.org/>), VMD (<http://www.ks.uiuc.edu/Research/vmd/>) or other specific programs. In our research, VMD was used.
